# Maternal complication related to instrumental delivery at Felege Hiwot Specialized Hospital, Northwest Ethiopia: a retrospective cross-sectional study

**DOI:** 10.1186/s13104-019-4530-7

**Published:** 2019-08-05

**Authors:** Shimeles Biru, Dagne Addisu, Simachew Kassa, Simachew Animen

**Affiliations:** 1 Department of Midwifery, College Of Medicine and Health Sciences, Debre Tabor University, Debre Tabor, Ethiopia; 2 Department of Midwifery, College Of Medicine and Health Sciences, Bahir Dar University, Bahir Dar, Ethiopia

**Keywords:** Maternal complication, Instrumental delivery, Northwest, Ethiopia

## Abstract

**Objective:**

The study aimed to determine proportion and risk factors for maternal complication related to forceps and vacuum delivery among mother who gave birth at Felege Hiwot Comprehensive Specialized Hospital (FHCSH).

**Results:**

Records of 406 mothers managed with instrumental vaginal delivery were reviewed and 97% of the reviewed card had complete documentation. The proportion of maternal complications related to instrumental delivery was 12.1%. A major complication of forceps assisted delivery was 2nd-degree perineal tear (7.4%), 3rd-degree perineal tear (1.5%), cervical tear (1.5%) and episiotomy extension (1%). However, the complication of vacuum-assisted vaginal delivery was only cervical tear (0.5%) and episiotomy extension (0.5%). Episiotomy during instrumental delivery reduce maternal complication by 86% [AOR = 0.14, 95% CI 0.07–0.3]. Forceps assisted vaginal delivery had 3.4 times more risk for maternal complication compared to vacuum-assisted vaginal delivery [AOR = 3.4, 95% CI 1.08–10.67] and the same is true for primiparity that primipara women who gave birth by the help of instrument had 3.5 times more risk for maternal complication compared to a multipara women [AOR = 3.5, 95% CI 1.26–9.98].

**Electronic supplementary material:**

The online version of this article (10.1186/s13104-019-4530-7) contains supplementary material, which is available to authorized users.

## Introduction

Instrumental vaginal delivery is applying obstetric forceps or vacuum device to effect in vaginal delivery of the fetus. These instrument-assisted deliveries are performed for the indication of maternal or fetal related condition and any event that threatens the mother or fetus but likely relieved by second-stage intervention [1–3].

Use of forceps and vacuum is common obstetrics practice in case of fetal distress or prolonged second stage of labor however they also carried a significant number of maternal complications like anal sphincter injuries.

In developed countries, a complication related to instrumental delivery is not significant as a result of advancement in skill on the management of instrumental delivery and accessibility of resources. However, developing countries like Ethiopia, mother and their newborn develop different degree of morbidity and even mortality due to instrumental delivery. Mostly those problems are avoidable if early interventions are undertaken [4, 5].

Complication due to instrumental delivery can be minor complication like laceration of vagina and perineum and major complication associated with traumatic haemorrhage, bladder injury and pelvic muscle injury [6].

In country like Ethiopia, there is insufficient evidence which shows on the magnitude and factor related to maternal complication of instrumental delivery. Knowledge on the magnitude of the problem and identification of possible risk factors for maternal complication related to instrumental delivery may reduce the maternal complication. Hence this study was aimed to determine the proportion of maternal complications related to instrumental vaginal delivery and its associated factors among mothers who gave birth at Felege Hiwot Comprehensive Specialized Hospital.

## Main text

### Methods

#### Study design and setting

A facility-based retrospective cross-sectional study was conducted at the maternity ward of Felege Hiwot Comprehensive Specialized Hospital. This hospital is located in Bahir Dar city which found approximately 565 km northwest of Addis Ababa.

The hospital has obstetrics, gynecology, pediatrics, internal medicine, ophthalmology and orthopedic surgery services. The labor ward gives services to around 612 deliveries per month. Department of Obstetrics and Gynecology had labor ward with seven beds in the first stage room, two delivery couches in the second stage room, four beds in the recovery unit and sixty-nine beds in the maternity ward along with two operating rooms.

##### Source population

Mothers who gave birth in Felege Hiwot Comprehensive Specialized Hospital from December 1, 2015 to November 30, 2017.

##### Study populations

Mothers who had an instrumental vaginal delivery in Felege Hiwot Comprehensive Specialized Hospital from December 1, 2015 to November 30, 2017.

### Sample size determination

The sample size was determined by using a single population proportion formula after considering the following assumptions:

The proportion of maternal complication related to instrumental vaginal delivery accounts for 59.8% from previous studies [7]. By taking 5% marginal error and 95% confidence interval, the required sample size was 369. After adding 10% for incomplete secondary data the final sample size was 406.

### Sampling procedure

The total number of women who gave birth in the hospital from December 1, 2015 to November 30, 2017 was 14,688. We identified and reviewed 841 chart, of which 21 charts were not available (lost) in the card room and through Systematic random sampling technique 406 charts were selected from 820 charts for this study (Additional file 1: Figure S1).

### Inclusion and exclusion criteria

#### Inclusion criteria

Medical records of all instrumental deliveries in the hospital from December 1, 2015 to November 30, 2017 were included in the study.

#### Exclusion criteria

Incompletely documented chart (chart which does not include all variable) and C/S delivery after failed instrument are excluded.

#### Socio-demographic, obstetric, enabling and instrumental delivery related variables

Socio-demographic, obstetric and other factors were examined as a potential predictor in this analysis. Socio-demographic factors include age and place of residence. Obstetric related factors include parity, number of pregnancy, gestational age, birth weight (BW), fetal presentation/position, station, current pregnancy type, frequency of ANC visit, duration of labour and Obstetric indications for IVD (prolonged second stage, non-reassuring fetal heart rate pattern, poor maternal effort, APH/abruption, after coming head of breech presentation, MSAF). Enabling factors are the type of hospital visit, instrument available and availability of electronic fetal monitoring (CTG used). Instrumental delivery related variables include types of instrument, the presence of episiotomy and type of episiotomy.

### Operational definitions

#### Maternal complication

Maternal complication related to instrumental delivery are the presence of at least one of the following (perineal tear, cervical laceration, vaginal laceration, episiotomy extension, traumatic hemorrhage (primary PPH), urinary retention, uterine rupture) [7].

### Data collection instruments

Data collection checklist was adapted and modified from different studies [7, 8].

### Data processing and analysis

Collected data were entered into EPI Info version 7 then exported to SPSS version 23 for analysis. Descriptive statistics used to describe the main features of the data. Bivariate analysis was done to identify candidate variable using p ≤ 0.2. Multivariate logistic regression was used to control the effect of confounder and variable with p ≤ 0.2 was included in multivariable logistic regression analysis. Finally, statistical significance declared at p-value < 0.05.

### Result

#### Socio-demographic characteristics

From four hundred six (406) mothers managed with instrumental vaginal delivery, three hundred ninety-seven (97%) of mother’s chart was completely documented. Two hundred forty-nine mothers (62.7%) were from urban areas. The mean age was 24.94 ± 4.7 years. Nearly half of the mothers were below 25 years 254 (64%) (Table 1).

**Table 1:** socio-demographic and obstetrics related characteristics of women who undergone instrumental deliveries at FCSH from December 1, 2015 to November 30, 2017

Variables	Frequency	Percent %
Age		
< 25	254	64.0
25–35	128	32.2
> 35	15	3.8
Place of residence		
Urban	249	62.7
Rural	148	37.3
Parity		
Primipara	280	70.5
Multipara	117	29.5
ANC visit		
Yes	385	97
No	12	3
Hospital visit type		
Referral	141	35.5
Direct visit	256	64.5
Gestational age		
Pre-term	7	1.8
Term	368	92.7
Post-term	22	5.5
Types of instrumental delivery		
Forceps delivery	332	83.6
Vacuum delivery	65	16.4
Type of instrumentation		
Mid	39	9.8
Low	215	54.2
Outlet	143	36.0
Birth weight		
< 2500	60	15.1
≥ 2500	337	84.9
Fetal position		
OA	334	84.2
OT	43	10.8
OP	20	5

#### Obstetric related characteristics

Two hundred eighty (70.5%) mothers were primipara. Three hundred eighty-five (97%) mothers had at least one ANC follow (Table 1).

#### Obstetric indication for instrumental delivery

Prolonged 2nd stage was the commonest indication for both forceps and vacuum-assisted delivery which accounts 110 (33%) and 29 (43.9%) respectively (Table 2).

**Table 2:** Obstetric indications for instrumental vaginal delivery at FHCSH from December 1, 2015 to November 30, 2017

Indication	Types of instrumental delivery	
	Forceps	Vacuum
Prolonged second stage	110 (33%)	29 (43.9%)
Fetal distress	100 (30%)	22 (33.3%)
Maternal exhaustion	99 (29.7%)	11 (16.7%)
PE/E	16 (4.7%)	3 (6.1%)
Other (GIIIMSAF, APH/abruption, prophylactic forceps)	7 (2.6%)	–

#### Proportion of maternal complication related to instrumental delivery

The proportion of maternal complication related to instrumental delivery was 12.1% [95% CI 9.3–15.3]. From the total complication, 91.7% was contributed by forceps-assisted delivery and only 8.3% of complications occur due to vacuum-assisted vaginal delivery. A major complication of forceps assisted delivery was 2nd-degree perineal tear (7.4%), 3rd-degree perineal tear (1.5%), cervical tear (1.5%), episiotomy extension (1%). complication of vacuum-assisted vaginal delivery was cervical tear (0.5%) and episiotomy extension (0.5%).

#### Factors associated with maternal complications related to instrumental vaginal delivery

Primiparity, episiotomy, absence of CTG, age of mother, hospital visit type, type of instrumental delivery, low instrumentation were variable identified in bivariate logistic regression analysis. However, after fitting those variables in multivariable logistic regression model; episiotomy, Primiparity and forceps-assisted instrumental vaginal delivery were a statistically significant association with maternal complication related to instrumental delivery.

Protective effect of episiotomy was shown for maternal complication related to instrumental delivery. Women who had an episiotomy during instrumental delivery were 86% lower maternal complication compared to women who didn’t have episiotomy [AOR = 0.14, 95% CI 0.07–0.3]. Furthermore, mother who had forceps delivery was 3.4 times more likely to develop maternal complication than mother who had vacuum delivery [AOR = 3.4, 95% CI 1.08–10.67]. Similarly being a primipara mother was about 3.5 times more risk for a complication of instrumental delivery than being multipara mother [AOR = 3.5, 95% CI 1.26–9.98] (Table 3).

**Table 3:** Factors associated with maternal complications related to instrumental vaginal deliveries in binary and multiple logistic regressions at FHCSH from December 1, 2015 to November 30, 2017

Variables	Maternal		COR (95% CI)	AOR (95% CI)	p-value
	Complication				
	No	Yes			
Is CTG available					
Yes	198	20	1	1	
No	151	28	0.55 [0.3–1.0]	0.68 [0.34–1.33]	0.266
Types of instrumental delivery					
Forceps	288	44	2.33 [0.81–6.73]	3.4 [1.08–10.67]	
Vacuum	61	4	1	1	0.034*
Does she had an episiotomy					
No	53	19	1	1	
Yes	296	29	0.27 [0.14–0.52]	0.14 [0.07–0.3]	0.000*
Does she had HTN					
No	327	38	1	1	
Yes	22	10	0.26 [0.15–2.84]	3.7 (0.4–31.)	0.23
Birth weight					
< 2500	50	10	1	1	
≥ 2500	299	38	0.64 [0.29–1.35]	1.41 [0.6–3.31]	0.43
Age of the mother					
< 25	216	38	1	1	
25–35	119	9	0.37 [0.17–0.81	2.5 [0.26–23.38]	0.44
> 35	14	1	0.4 [0.05–3.08]	0.8 [0.08–7.76]	0.85
Parity					
Primipara	238	42	3.3 [1.34–7.9]	3.5 [1.26–9.98]	0.017*
Multipara	111	6	1	1	
ANC follow up					
No	9	3	0.4 [.104–1.52]	2.82 [0.6–13.14]	0.186
Yes	340	45	1	1	
Hospital visit type					
Referral	117	24	1.98 [1.1–3.64]	1.58 [0.81–3.06]	0.17
Direct visit	232	24	1	1	
Fetal station					
Mid	37	2	1	1	
Low	188	27	0.35 [.08–1.58]	0.2 [0.04–1.05]	0.06
Outlet	124	19	0.94 [0.5–1.76]	1.02 [0.5–2.06]	0.96

* p-value < 0.05

### Discussion

Overall maternal complication related to instrumental delivery was 12.1%. This finding was consistent with different other studies. Mothers who had the procedure developed complications like second and third-degree perineal tear as well as episiotomy extension [9–11]. However, this finding was in contrast with other study finding like study in United States and England [12, 13]. This can be due to the population described in this study is highly different in wealth quintile and service quality provided.

There is a significant difference in maternal complications between women delivered by forceps as compared to those delivered by vacuum. Mothers who had forceps delivery were 3.4 times more likely to develop maternal complication than those mothers who had vacuum delivery. These findings are in accordance with the Cochrane database review study that maternal morbidity was less in vacuum extraction compared to forceps delivery. This is evidenced by vacuum extraction was associated with less pain at delivery and less likely to cause serious injury on the mother [14, 15]. Recently vacuum extraction is most commonly used when an instrument is needed to facilitate vaginal delivery, and also this is observed in different other studies. Forceps deliveries appear to have lost their favour. This shift in practice may have been influenced both by the evidence of dramatically reduced maternal trauma with vacuum extraction compared with forceps delivery. However, others showed that perineal damage like second and third-degree lacerations had no difference between the two methods [16, 17].

Mothers who had episiotomy were about 86% less risk for maternal complication of instrumental delivery than those who had no episiotomy. This is also true that mediolateral episiotomy protected significantly for anal sphincter damage in both vacuum extraction and forceps delivery. Mediolateral episiotomy in operative vaginal delivery strongly protects the occurrence of anal sphincter lesions [18, 19].

Primipara mother was about 3.5 times more likely risk for a complication of instrumental delivery than multipara mother. A possible explanation for this may be due to a higher tendency to second stage delays in primigravida mother. Even though the exact mechanism is not justified primipara women had a high risk for perineal injuries [18].

### Conclusions

Maternal complication related to instrumental vaginal delivery is high in Felege Hiwot Comprehensive Specialized Hospital. Forceps assisted vaginal delivery and primiparity were a significant risk factor associated with maternal complication related to instrumental delivery. However, episiotomy found to be strongly protective for maternal complication during instrumental delivery so liberal use of episiotomy during instrumental delivery is encouraged.

## Limitation

- This study shares the limitations of cross-sectional studies and hence may not be possible to establish a temporal relationship between maternal complication due to instrumental vaginal delivery and explanatory variables.
- It was a retrospective study; important variables like socio-demographic status, body mass index of the mother and sequential use of instrument were not addressed in this study.
- The study was conducted in a single hospital; the results might not be a representative of other institutions.

## Additional file

**Additional file 1: Figure S1.** Schematic presentation of sampling technique.

## Data Availability

The datasets used in this study are available from the corresponding author on reasonable request.

## Abbreviations

ANC: antenatal care
APH: ante partum haemorrhage
CS: caesarean section
FHCSH: Felege Hiwot Comprehensive Specialized Hospital
IVD: instrumental vaginal delivery
OVD: operative vaginal delivery
PPH: postpartum haemorrhage
