# Supplementary material for: Maternal complication related to instrumental delivery at Felege Hiwot Specialized Hospital, Northwest Ethiopia: a retrospective cross-sectional study
Source: BMC Res Notes. 2019 Aug 5;12:482. doi: 10.1186/s13104-019-4530-7 (PMC6683480; doi:10.1186/s13104-019-4530-7)
Supplement: Supplementary file 1 — Additional file 1: Figure S1. Schematic presentation of sampling technique. [file 13104_2019_4530_MOESM1_ESM.docx]

# SAMPLING TECHNIQUE

During the study period a total of 14688 delivery services were registered in the maternity ward including C/S, IVD and spontaneous vaginal delivery. Through Medical Registration Number(MRN) 841 mother’s chart was named as instrumental delivery in the maternity ward delivery registration log book but only 820 chart’s was collected and reviewed in the hospital’s card room, the remaining 21 charts were lost.

**Figure S1: Schematic presentation of sampling technique**
